# Wedge resection is equal to segmental resection for pulmonary typical carcinoid patients at localized stage: a population-based analysis

**DOI:** 10.7717/peerj.7519

**Published:** 2019-09-10

**Authors:** Tao Yan, Kai Wang, Jichang Liu, Yukai Zeng, Fenglong Bie, Guanghui Wang, Jiajun Du

**Affiliations:** 1 Institute of Oncology, Shandong Provincial Hospital Affiliated to Shandong University, Jinan, China; 2 Department of Healthcare Respiratory, Shandong Provincial Hospital Affiliated to Shandong University, Jinan, China; 3 Department of Thoracic Surgery, Shandong Provincial Hospital Affiliated to Shandong University, Jinan, China

**Keywords:** Pulmonary typical carcinoid, Wedge resection, Segmental resection, Prognosis

## Abstract

**Background:**

Medical institutions worldwide have not reached a consensus on what surgery is the most advisable for pulmonary typical carcinoid (TC) patients at the localized stage. This research focuses on exploring whether wedge resection or segmental resection is the superior option.

**Methods:**

The demographic and clinical information of 1,887 TC patients diagnosed at the localized stage from 2004 to 2015 was collected from the Surveillance, Epidemiology, and End Results (SEER) Program. Patient prognosis was evaluated by KM curves. The chi-square test was used to examine the variation between different groups that would be eliminated by propensity score matching (PSM). Univariate and multivariate Cox proportional hazard model analyses were used to evaluate prognostic values of relative factors.

**Results:**

The prognosis of TC was the most favorable for patients suffering from pulmonary squamous cell carcinoma (SCC), adenocarcinoma (ADC), and pulmonary carcinoids (PCs). The choice to have surgery, not the type of surgery chosen, was the most significant independent prognostic factor correlated with overall survival (OS) and lung cancer-special survival (LCSS). The prognostic result of the comparison between wedge resection and segmental resection was not statistically significant before or after PSM. In subgroup analysis, the inference still held.

## Introduction

Typical carcinoid (TC) is one of four major pathological classifications of neuroendocrine tumor (NET) of the lung categorized by the World Health Organization (WHO). The others are atypical carcinoid (AT), large-cell neuroendocrine carcinoma (LCNEC), and small-cell lung cancer (SCLC) (Caplin et al., 2015). Data shows that bronchopulmonary or lung NETs constitute approximately 25 percent of carcinoma of the lungs, while the prevalence of primary NETs is 20–25% (Ramirez et al., 2017; Yao et al., 2008). Compared with other types of NET, the incidence of TC and AT is lower and accounts for 1–2% of pulmonary malignant tumor (Chen, Travis & Krug, 2006; Travis, 2010). Another study indicates that there are 10 times more TC than AT patients (Gosain et al., 2018; Rekhtman, 2010). Until now, surgery has been the main treatment for TC, the low-grade lung NET, at the localized stage (Wolin, 2015; Wolin, 2017). The recommendation of adjuvant therapy, especially radiation and chemotherapy, remains controversial (Wolin, 2017).

For early stage patients with operable TC, there is no general agreement on the optimal operation mode (Fox et al., 2013; Mezzetti et al., 2003; Raz et al., 2015; Rea et al., 2007). Lobectomy and sublobectomy (wedge resection and segmental resection) are the major surgical options for localized diseases. Traditionally, anatomic resection (lobectomy and segmental resection) is considered the best option for patients with peripheral lung tumors, while segmentectomy and wedge resection are widely used for patients who have limited pulmonary function (Caplin et al., 2015). Recently, Taher Abu Hejleh et al. (Furqan et al., 2018), after collecting and researching the SEER data, suggested that the prognoses for lobectomy and sublobectomy were comparable. Some studies have shown segmental resection to be superior to wedge resection in liver cancer and non-small cell lung cancer (NSCLC) (El-Sherif et al., 2007; DeMatteo et al., 2000). Other studies found no differences between wedge resection and segmental resection in lung neoplasm (Altorki et al., 2016; Romano & Mark, 1992). However, currently no research compares the two operations, wedge resection and segmental resection, which constitute the sublobectomy, in TC patients. Although both surgeries are sublobectomies, wedge resection causes a smaller operative wound than segmental resection.

Now that surgery is the main choice of treatment for TC patients, and given that other therapeutic methods are not powerful, the smaller operative wound is a major consideration after guaranteeing the survival rate. The objective of this research is to determine if there is a difference between wedge resection and segmental resection for early stage TC patients.

## Materials & Methods

### Data source

We have been given access to the SEER databases in October 2018 with the “numbered” SEER*Stat account(12991-Nov2017). The retrospective research collected the data from the Surveillance, Epidemiology and End Results (SEER) by using SEER*Stat 8.3.5 in December 2018. The inclusion criteria were as follows: (I) TC patients (code 8240) at the localized stage; (II) patients treated via no surgery at the primary site (code 00), wedge resection (code 21), segmentectomy (code 22), or lobectomy (code 33); (III) patients with only one primary tumor. The exclusion criteria included: (I) diagnosis before 2004, (II) survival of less than one month or unknown, (III) lung cancer-special survival (LCSS) missing, (IV) tumor size unknown, (V) scope of regional lymph node surgery unknown, (VI) radiation therapy unknown, and (VII) regional, distant, or unknown summary stage.

### Variates

Demographic information for patients included patient ID, age, gender, and race. Age at diagnosis was divided into two groups, under 70 and 70 or older. Race was categorized into white and other. Variables collected for the research included pathological data, treatment, and follow-up information, such as laterality, surgery, procedure used, tumor size, scope of regional lymph node surgery, radiation, chemotherapy, summary stage, differentiation grade, overall survival status, LCSS, and survival month.

### Statistical analyses

Unbalanced distribution of variables between groups was evaluated by chi-square test. A KM curve was generated to assess survival and a log-rank test was used to evaluate survival discrepancy. Univariate and multivariate Cox proportional hazards regressions were conducted to evaluate the effects of involved factors on prognosis, acquire hazards ratio, and generate a 95% confidence interval (HR and 95% CI). Propensity score matching (PSM) of 1:1 was used to eliminate the differences of variable components between wedge and segmental resection, and the caliper was 0.01. Statistical analysis was performed with SPSS 24.0 software (SPSS Inc, Chicago, IL). Significance was set at *P* of less than 0.05.

## Result

### Prognosis for carcinoid, squamous cell cancer (SCC), and adenocarcinoma (ADC) in lung

**Figure 1:**
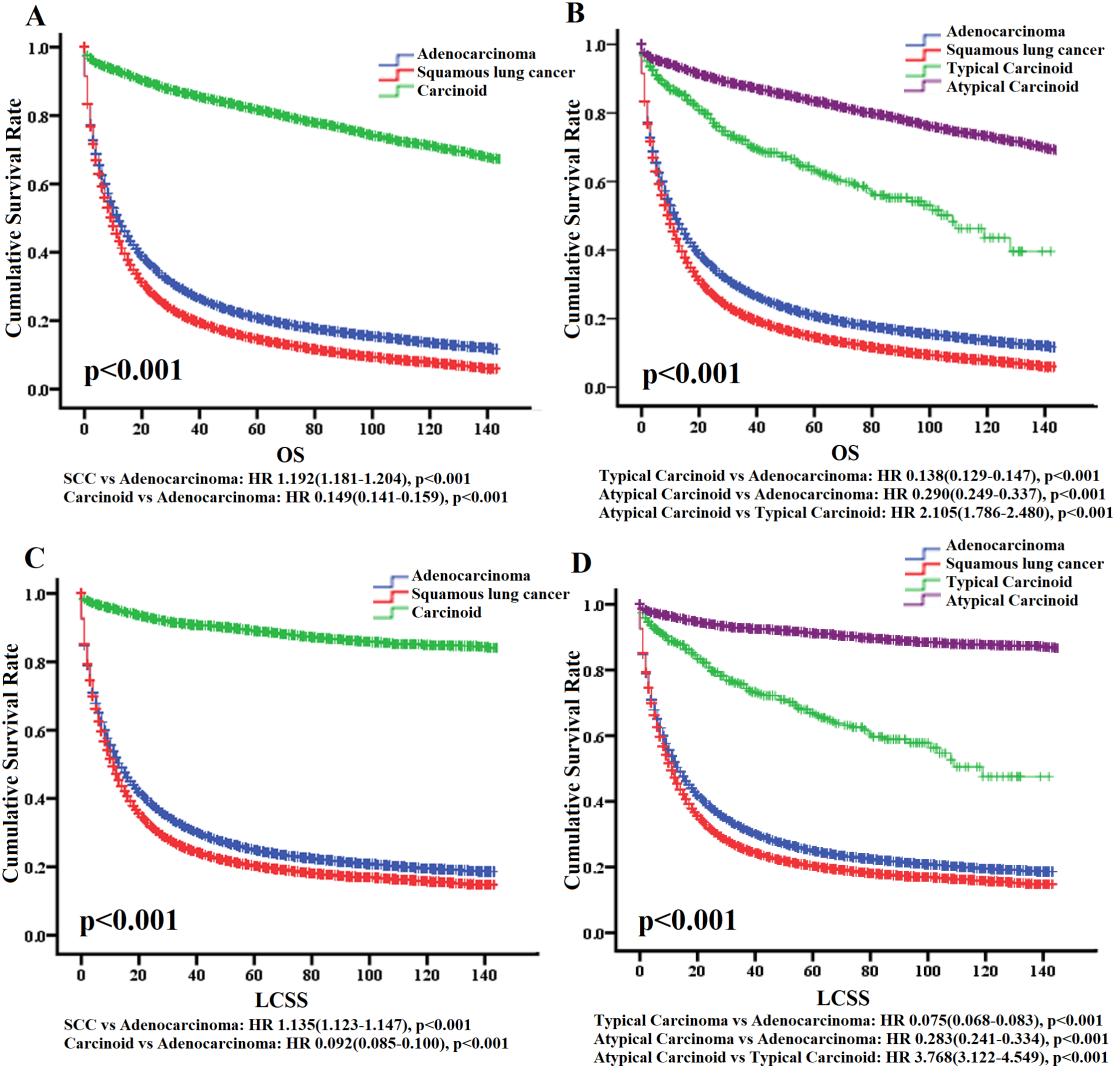
OS and LCSS for ADC, SCC and carcinoid patients evaluated using KM plots. Overall survival and lung cancer-special survival among adenocarcinoma, squamous lung cancer and carcinoid (A, C). Overall survival and Lung cancer-special survival among adenocarcinoma, squamous lung cancer, typical carcinoid and atypical carcinoid (B, D). HR is from Cox regression analysis. *P* value is from a log-rank test. HR, hazard ratio; OS, overall survival; LCSS, Lung cancer-special survival.

Presently squamous cell cancer and adenocarcinoma are common forms of lung cancer, accounting for 78% of this disease. Lung carcinoid is composed of typical carcinoid and atypical carcinoid. To acquire general prognosis information about TC patients, we compared the cumulative survival rate for lung carcinoid and its components with SCC and ADC. The clinical information was recorded in Table 1. Obviously, carcinoid has the more favorable prognosis compared with SCC and ADC in overall survival (OS) and lung cancer-specific survival (LCSS) (Fig. 1), as follows: hazard ratio (HR) = 0.149 (0.141–0.519), *P* < 0.001 of carcinoid versus ADC in OS; HR = 0.092 (0.085–0.100), *P* < 0.001 of carcinoid versus ADC in LCSS; HR=1.192 (1.181–1.204), *P* < 0.001 of SCC versus ADC in OS; HR = 1.135 (1.123–1.147), *P* < 0.001 of SCC versus ADC in LCSS.

**Table 1:** Baseline Information for comparison among Adenocarcinoma, SCC and Carcinoid.

	Adenocarcinoma	SCC	Carcinoid	AC	TC
Race						
White	133,061	66,319	4,666	4,183	483
Black	19,659	10,057	381	344	37
Others	15,546	4,311	197	19	178
Gender						
Female	87,558	29,751	3,574	350	3,224
Male	80,708	50,936	1,670	189	1,481
Primary Site					
Main Bronchus	4,914	5,190	286	30	256
Upper Lobe	84,247	42,587	1,540	171	1,369
Middle Lobe	8,221	2,833	897	88	809
Lower Lobe	44,029	22,265	2,117	201	1,916
Others	26,855	7,812	404	49	355
Laterality					
Left	63,614	33,873	2,065	215	1,850
Right	95,605	44,602	3,069	314	2,755
Others	9,047	2,212	110	10	100
Grade						
I	15,316	1,642	1,767	60	1,707
II	32,248	21,232	488	175	313
III	39,710	27,415	39	11	28
IV	1,457	574	15	5	10
Others	79,535	29,824	2,935	288	2,647
TNM Stage						
I	33,843	17,665	1,936	240	1,696
II	6,339	5,573	400	56	344
III	34,607	24,946	301	112	189
IV	80,900	27,311	338	104	234
Others	12,577	5,192	2,269	27	2,242
T						
0	895	189	0	0	0
1	34,263	11,815	1,703	217	1,486
2	42,925	27,268	724	172	552
3	6,797	7,486	268	13	255
4	61,538	26,729	259	99	160
Others	21,848	7,200	2,290	38	2,252
N						
0	63,527	31,446	2,504	303	2,201
1	13,234	7,844	247	72	175
2	53,635	28,058	264	126	138
3	20,096	8,247	38	15	23
Others	17,774	5,092	2,191	23	2,168
M						
0	77,237	50,072	2,785	418	2,367
1	80,900	27,311	338	104	234
Others	10,129	3,304	2,121	17	2,104
Summary Stage					
Localized	31,653	15,415	3,346	225	3,121
Regional	33,274	25,968	1,072	178	894
Distant	99,878	37,202	675	119	556
Others	3,461	2,102	151	17	134
Surgery						
No	120,379	61,452	1,052	127	925
Sublobar Resection	9,597	3,116	1,128	95	1,033
Lobectomy/Bilobectomy	35,236	12,992	2,790	280	2,510
Pneumonectomy	1,559	2,008	186	29	157
Others	1,495	1,119	88	8	80
Chemotherapy					
No/Unknown	93,973	46,482	4,953	406	4,547
Yes	74,293	34,205	291	133	158
Radiotherapy					
No/Unknown	109,686	43,866	4,973	458	4,515
Yes	58,580	36,821	271	81	190
Overall Survival Rate					
3-year Survival (%)	30.81	22.67	87.88	73.28	89.38
5-year Survival (%)	22.78	16.01	83.03	64.50	84.84
10-year Survival (%)	14.81	8.56	72.43	44.30	74.49
Lung Cancer Specific Survival Rate					
3-year Survival (%)	34.29	27.55	92.03	76.70	93.60
5-year Survival (%)	27.01	21.92	89.89	67.84	92.01
10-year Survival (%)	21.16	17.01	85.70	48.14	88.45

**Notes.**

SCC Squamous lung carcinoma AC Atypical Carcinoid TC Typical Carcinoid

Furthermore, TC has a better prognosis than AT in OS and LCSS, as follows: HR = 2.105 (1.786–2.480), *P* < 0.001 of AT versus TC in OS; HR=3.768 (3.122–4.549), *P* < 0.001 of AT versus TC in LCSS.

In conclusion, typical carcinoid has a more favorable prognosis than the other three types of lung cancers.

### Baseline information of TC patients from SEER database

There were 1,887 TC patients in our cohort: 235 patients with no surgery, 465 undergoing wedge resection, 113 receiving segmental resection, and 1,074 going under the lobectomy. Some demographic and clinical information, such as age, gender, race, etc., was been recorded (Table 2). Notably, 71.8% patients were female and most patients were white. Compared with the no surgery group, the percentages of old patients (age ≥ 70 years) are less than 30% of the other three surgery groups: 58.3% in no surgery, 27.1% in wedge resection, 15.9% in segmental resection, and 18.1% in lobectomy. Thus, it can be seen that age may be an important factor in choosing a treatment option. In addition, the number of female patients is much larger than that of male patients no matter what treatment they receive. As for the factor “scope of the lymph node,” wedge resection results in less regional lymph node resection compared to segmental resection or lobectomy: the percentage of no regional lymph node resection is 68.6% in wedge resection and 38.9% in segmental resection.

**Table 2:** Baseline characteristics of all TC patients.

Characteristic	No surgery	Wedge	Segmental	Lobectomy	Total
	(*n* = 235)	(*n* = 465)	(*n* = 113)	(*n* = 1074)	(*n* = 1887)
Age					
<70y	98 (41.7%)	339 (72.9%)	95 (84.1%)	880 (81.9%)	1412 (74.8%)
≥70y	137 (58.3%)	126 (27.1%)	18 (15.9%)	194 (18.1%)	475 (25.2%)
Gender					
Female	172 (73.2%)	355 (76.3%)	80 (70.8%)	738 (68.7%)	1345 (71.3%)
Male	63 (26.8%)	110 (23.7%)	33 (29.2%)	336 (31.3%)	542 (28.7%)
Race					
White	201 (85.5%)	419 (90.1%)	102 (90.3%)	979 (91.2%)	1701 (90.1%)
Black	25 (10.6%)	27 (5.8%)	8 (7.1%)	54 (5.0%)	114 (6.0%)
Others	9 (3.9%)	19 (4.1%)	3 (2.6%)	41 (3.8%)	72 (3.9%)
Grade					
Well/Moderate	76 (32.3%)	194 (41.7%)	52 (46.0%)	571 (53.2%)	893 (47.3%)
Poor/Undifferentiated	1 (0.4%)	3 (0.6%)	1 (0.9%)	3 (0.3%)	8 (0.4%)
Unknown	158 (67.3%)	268 (57.7%)	60 (53.1%)	500 (46.5%)	986 (52.3%)
Laterality					
Right	146 (62.1%)	261 (56.1%)	43 (38.1%)	648 (60.3%)	1098 (58.2%)
Left	89 (37.9%)	204 (43.9%)	70 (61.9%)	426 (39.7%)	789 (41.8%)
Tumor size					
T1 (≤3 cm)	184 (78.3%)	453 (97.4%)	105 (92.9%)	904 (84.2%)	1646 (87.2%)
T2 (≤5 cm, <3 cm)	38 (16.2%)	11 (2.4%)	7 (6.2%)	138 (12.8%)	194 (10.3%)
T3 (≤7 cm, >5 cm)	6 (2.5%)	– –	1 (0.9%)	20 (1.9%)	27 (1.4%)
T4 (>7 cm)	7 (3.0%)	1 (0.2%)	– –	12 (1.1%)	20 (1.1%)
Scope of regional lymph node					
0	235 (100%)	319 (68.6%)	44 (38.9%)	4 (0.4%)	602 (31.9%)
1–3	– –	88 (18.9%)	29 (25.7%)	166 (15.5%)	283 (15.0%)
>3	– –	58 (12.5%)	40 (35.4%)	904 (84.1%)	1002 (53.1%)
Radiation					
No	209 (89.9%)	462 (99.4%)	112 (99.1%)	1068 (99.4%)	1851 (98.1%)
Yes	26 (11.1%)	3 (0.6%)	1 (0.9%)	6 (0.6%)	36 (1.9%)
Chemotherapy					
No	232 (98.7%)	464 (99.8%)	112 (99.1%)	1068 (99.4%)	1876 (99.4%)
Yes	3 (1.3%)	1 (0.2%)	1 (0.9%)	6 (0.6%)	11 (0.6%)

### Prognosis for TC patients treated with no surgery, wedge resection, segmental resection, and lobectomy

**Figure 2:**
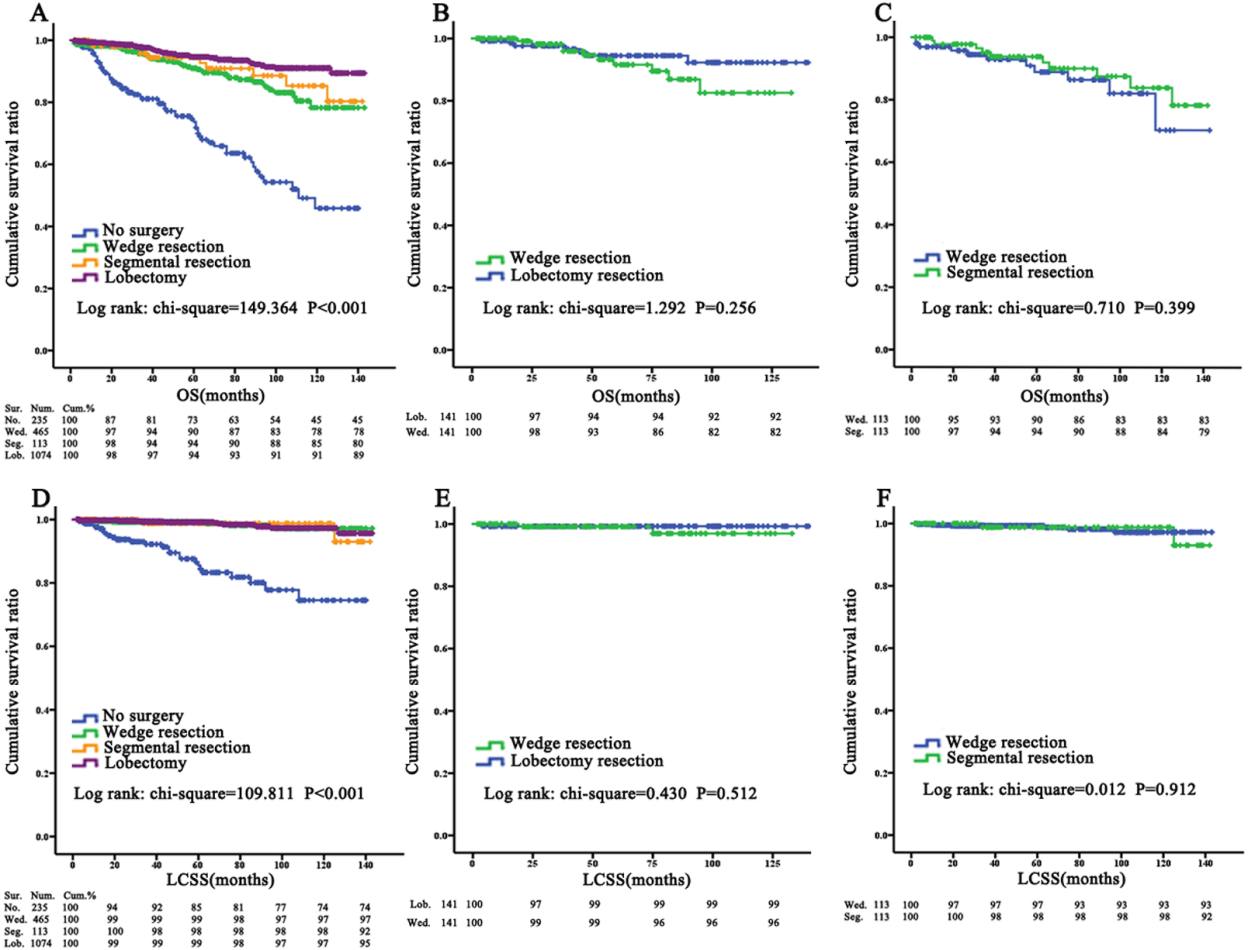
OS and LCSS for pulmonary TC patients evaluated using KM plots. Overall survival (A) and lung cancer-special survival (D) among no surgery (*n* = 235), wedge resection (*n* = 465), segmental resection (*n* = 113) and lobectomy (*n* = 1,074) TC patients at localized stage form SEER project. Overall survival (B) and lung cancer specific survival (E) in wedge resection versus lobectomy after PSM. Overall survival (C) and lung cancer specific survival (F) in wedge resection versus segmental resection after PSM. The difference of OS between two operations was eliminated after PSM: *P* = 0.256. *P* value is from a log-rank test. OS, overall survival; LCSS, Lung cancer-special survival; No., no surgery; Wed., wedge resection; Seg., segmental resection; Lob., lobectomy.

The influence of surgery and surgery type on overall survival (OS) and lung cancer-specific survival (LCSS) for pulmonary TC patients were evaluated using KM curves (Figs. 2A, 2D). There were significant differences between the group with no surgery and the other three groups, both in OS and LCSS: The 5-year survival rate of the no surgery, wedge resection, segmental resection, and lobectomy, respectively, were 73%, 90%, 94%, and 94% for OS (*P* < 0.001). There is an even greater gap between patients with no surgery and groups who underwent surgery when the 10-year survival rate is examined.

**Figure 3:**
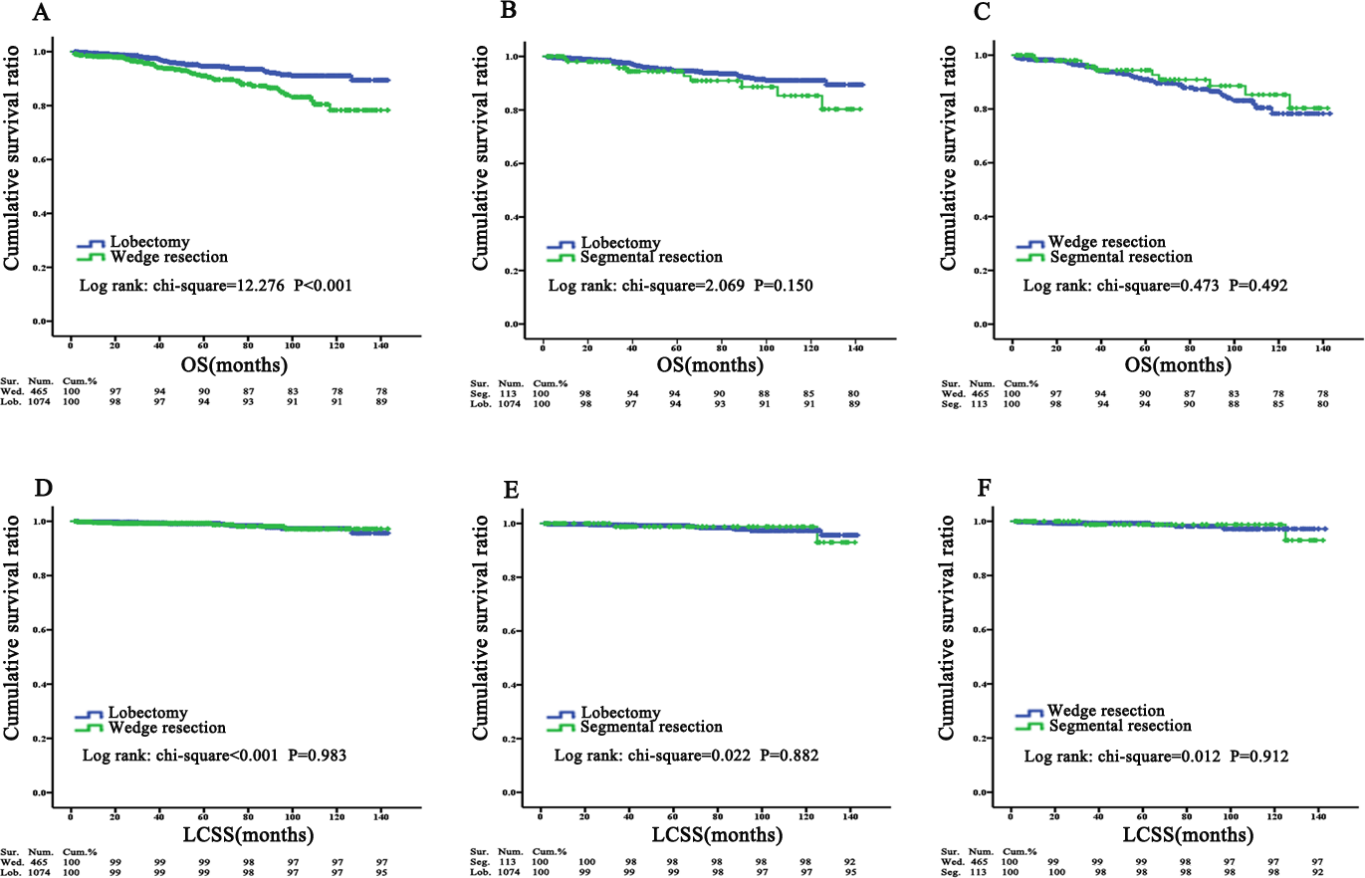
Three surgery groups were assessed as pairs with KM curves. Overall survival and lung cancer specific survival in wedge resection versus lobectomy, segmental resection versus lobectomy, wedge resection versus segmental resection. There was no significant difference between two surgical options in OS except wedge versus lobectomy: (A) wedge resection versus lobectomy, *P* < 0.001; (B) segmental resection versus lobectomy, *P* = 0.150; (C) wedge resection versus segmental resection, *P* = 0.492. There was no significant difference between two surgical options in LCSS: (D) wedge resection versus lobectomy, *P* = 0.983; (E) segmental resection versus lobectomy, *P* = 0.882; (F) wedge resection versus segmental resection, *P* = 0.912. OS, overall survival; LCSS, Lung cancer-special survival; No., no surgery; Wed., wedge resection; Seg., segmental resection; Lob., lobectomy.

For further research about the impact of surgery type, the three groups were defined here as pairs to be assessed with KM curves (Fig. 3). Although the comparison between lobectomy and wedge resection indicated that the difference in patient prognoses was statistically significant for OS (*P* < 0.001), the propensity score match (PSM) eliminated this diversity between those groups for OS (*P* = 0.256) (Figs. 2B, 2E). The results of other contrast groups showed comparable prognoses for both OS and LCSS (*P* > 0.05).

### Univariate and multivariate COX regression analyses of TC patients in the localized stage

Relative risk factors for OS were analyzed using univariate and multivariate COX regression hazard models (Table 3). The factors whose *P* < 0.05, both in univariate and multivariate analysis, are: age (*P* < 0.001 in multivariate analysis), gender (*P* = 0.037 in multivariate analysis), and surgery (*P* < 0.001 in multivariate analysis). For OS, age, gender, and surgery were independent prognostic factors with HR as follows: 3.889 (95% CI [2.536–5.965], ≥70 y versus <70 y), 1.574 (95% CI [1.028–2.410], female versus male), 0.520 (95% CI [0.317–0.853], wedge resection versus no surgery, *P* = 0.008), 0.259 (95%CI [0.095–0.706], segmental resection versus no surgery, *P* < 0.001), 0.159 (95% CI [0.059–0.427], lobectomy versus no surgery, *P* < 0.001).

**Table 3:** Univariate and multivariate Cox regression analyses according to OS for TC patients.

Variables in the equation	Univariate		Multivariate	
	*P*	Hazard ratio (95% CI)	*P*	Hazard ratio (95% CI)
Age				
<70y	–	–	–	–
≥70y	^*^<0.001	4.553 (3.358–6.173)	^*^<0.001	3.889 (2.536–5.965)
Gender				
Female	–	–	–	–
Male	0.008	1.521 (1.116–2.073)	0.037	1.574 (1.028–2.410)
Race				
White	0.050	–	0.056	–
Black	0.033	1.753 (1.047–2.936)	0.056	2.058 (0.980–4.321)
Others	0.272	0.527 (0.168–1.652)	0.166	0.247 (0.034–1.782)
Grade				
Well/Moderate	^*^<0.001	–	0.176	–
Poor/Undifferentiated	0.011	6.285 (1.522–25.950)	0.063	3.992 (0.930–17.137)
Unknown	^*^<0.001	2.172 (1.449–3.255)	0.902	1.035 (0.600–1.785)
Laterality				
Right	–	–	–	–
Left	0.908	0.982 (0.724–1.333)	–	–
Tumor size				
T1 (≤1 cm)	0.112	–	–	–
T2 (≤2 cm, >1 cm)	0.309	0.811 (0.543–1.213)	–	–
T3 (≤3 cm, >2 cm)	0.092	0.660 (0.407–1.070)	–	–
T4 (>3cm)	0.533	1.168 (0.717–1.902)	–	–
Scope of regional lymph node				
*n* = 0	^*^<0.001	–	0.357	–
*n* > 0, *n* ≤ 3	^*^<0.001	0.346 (0.212–0.566)	0.960	0.974 (0.357–2.659)
*n* > 3	^*^<0.001	0.305 (0.217–0.428)	0.225	1.763 (0.706–4.403)
Surgery				
No surgery	^*^<0.001	–	0.001	–
Wedge resection	^*^<0.001	0.281 (0.192–0.412)	0.008	0.520 (0.317–0.853)
Segmental resection	^*^<0.001	0.228 (0.117–0.444)	^*^<0.001	0.259 (0.095–0.706)
Lobectomy	^*^<0.001	0.140 (0.097–0.202)	^*^<0.001	0.159 (0.059–0.427)

**Notes.**

* *p* < 0.05, statistically significant.

The hazard study for LCSS was completed in the same way (Table 4). Data showed that age (*P* = 0.008 in multivariate analysis), tumor size (*P* = 0.012 in multivariate analysis), and surgery (*P* = 0.004 in multivariate analysis) are independent prognostic factors whose *P* < 0.05 both in univariate and multivariate analysis. As for LCSS, the HR of independent prognostic factors is as follows: 2.261 (95% CI [1.232–4.149], ≥70 y versus <70 y), 0.167 (95%CI [0.061–0.461], wedge resection versus no surgery, *P* = 0.001), 0.197 (95% CI [0.040–0.967], segmental resection versus no surgery, *P* = 0.045), 0.158 (95% CI [0.026–0.951], lobectomy versus no surgery, *P* = 0.044). The independent prognostic factors for both OS and LCSS were age and surgery.

**Table 4:** Univariate and multivariate Cox regression analyses according to LCSS for TC patients.

Variables in the equation	Univariate		Multivariate	
	*P*	Hazard ratio (95% CI)	*P*	Hazard ratio (95% CI)
Age				
<70y	–	–	–	–
≥70y	^*^<0.001	4.112 (2.341–7.225)	^*^0.008	2.261 (1.232–4.149)
Gender				
Female	–	–	–	–
Male	0.362	1.315 (0.730–2.368)	–	–
Race				
White	0.348	–	–	–
Black	0.151	1.753 (1.047–2.936)	–	–
Others	0.755	0.527 (0.168–1.652)	–	–
Grade				
Well/Moderate	0.086	–	–	–
Poor/Undifferentiated	^*^0.027	9.928 (1.301–75.743)	–	–
Unknown	0.756	1.143 (0.492–2.655)	–	–
Laterality				
Right	–	–	–	–
Left	0.641	1.143 (0.651–2.007)	–	–
Tumor size				
T1 (≤1 cm)	^*^<0.001	–	^*^0.012	–
T2 (≤2 cm, >1 cm)	0.924	0.955 (0.371–2.463)	0.663	0.806 (0.305–2.127)
T3 (≤3 cm, >2 cm)	0.536	1.369 (0.506–3.703)	0.670	1.255 (0.441–3.574)
T4 (>3 cm)	^*^0.005	3.853 (1.519–9.774)	^*^0.060	2.586 (0.960–6.968)
Scope of regional lymph node				
*n* = 0	^*^<0.001	–	0.357	–
*n* > 0, *n* ≤ 3	^*^0.003	0.169(0.052–0.552)	0.960	0.974 (0.357–2.659)
*n* > 3	^*^<0.001	0.244(0.129–0.465)	0.225	1.763 (0.706–4.403)
Surgery				
No surgery	^*^<0.001	–	^*^0.004	–
Wedge resection	^*^<0.001	0.089(0.037–0.216)	^*^0.001	0.167 (0.061–0.461)
Segmental resection	^*^0.002	0.106(0.025–0.448)	^*^0.045	0.197 (0.040–0.967)
Lobectomy	^*^<0.001	0.089(0.047–0.170)	^*^0.044	0.158 (0.026–0.951)

**Notes.**

* *p* < 0.05, statistically significant.

### Prognosis of TC patients who underwent wedge resection or segmental resection before and after PSM

According to the selection criterion, 465 patients in the cohort received wedge resection and 113 underwent segmental resection. A distinctly unequal distribution of factors existed between these two groups (Table 5). To reach a more objective conclusion, factors such as age, laterality, tumor size, and scope of regional lymph node must be balanced. Compared with wedge resection, segmental resection was more often offered to people of older age, with larger tumor sizes, who had a larger region of lymph node resected.

**Table 5:** Baseline characteristics for patients whom underwent wedge resection/segmentectomy before and after PSM.

Characteristic	Before PSM			After PSM		
	Wedge R	Segmental R	*P*	Wedge R	Segmental R	*P*
	(*n* = 465)	(*n* = 113)		(*n* = 100)	(*n* = 100)	
Age			^*^0.015			1.000
<70y	339	95		83	82	
≥70y	126	18		17	18	
Gender			0.226			0.629
Female	355	80		76	72	
Male	110	33		24	28	
Race			1.000			0.822
White	419	102		88	90	
Others	46	11		12	10	
Grade			0.611			0.352
Well/Moderate	172	45		41	39	
Poor/Undifferentiated	25	8		2	6	
Unknown	268	60		57	55	
Laterality			^*^0.001			1.000
Right	261	43		42	41	
Left	204	70		58	59	
Tumor size			^*^<0.001			0.251
T1 (≤1 cm)	159	30		41	30	
T2 (≤2 cm, >1 cm)	253	50		38	43	
T3 (>2 cm)	53	33		21	27	
Scope of regional lymph node			^*^<0.001			0.845
0	319	44		37	41	
1–3	88	29		31	29	
>3	58	40		32	30	
Radiation			0.058			1.000
No	462	112		100	99	
Yes	3	1		0	1	
Chemotherapy			0.353			1.000
No	464	112		100	99	
Yes	1	1		0	1	

**Notes.**

* *p* < 0.05, statistically significant.

To avoid being influenced by factors’ disproportionate distribution and data bias, we used PSM (an analysis function of the software, Statistical Package for the Social Sciences) to acquire an adequate sample set. First, we found the unbalanced variates by *t*-test. Then we chose the PSM to solve those imbalances by matching analogical cases from two groups. After 1:1 PSM, 100 patients who underwent wedge resection and 100 patients with segmental resection were involved in the final research. Meanwhile, all factors, including age, laterality, tumor size, were balanced to some extent (Table 5).

**Table 6:** Univariate Cox regression analyses according to OS and LCSS for TC patients whom underwent wedge and segmental resection.

Variables in the equation	OS		LCSS	
	*P*	Hazard ratio (95% CI)	*P*	Hazard ratio (95% CI)
Age				
<70y	–	–	–	–
≤70y	^*^<0.001	4.141 (2.430–7.058)	*0.021	5.428 (1.296-22.728)
Gender				
Male	–	–	–	–
Female	0.093	1.612 (0.924–2.812)	0.915	1.092 (0.220–5.430)
Race				
White	–	–	–	–
Others	0.581	0.751 (0.271–2.077)	0.575	0.331 (0.007–15.723)
Grade				
Well/Moderate	0.872	–	0.681	–
Poor/Undifferentiated	0.604	0.676 (0.154–2.963)	0.488	2.350 (0.210–26.312)
Unknown	0.830	0.935 (0.508–1.722)	0.902	0.899 (0.167–4.847)
Laterality				
Right	–	–	–	–
Left	0.812	1.066 (0.628–1.810)	0.143	3.311 (0.668–16.417)
Tumor size				
T1 (≤1 cm)	0.456	–	0.682	–
T2 (≥2 cm, >1 cm)	0.377	0.776 (0.443-1.361)	0.653	0.693 (0.140–3.438)
T3 (≤2 cm)	0.258	0.595 (0.242–1.463)	0.638	1.537 (0.257–9.202)
Scope of regional lymph node				
*n* ≤ 3	–	–	–	–
*n* > 3	0.698	0.845 (0.361–1.979)	0.297	2.366 (0.470–11.917)
Surgery				
Wedge resection	–	–	–	–
Segmental resection	0.493	0.786 (0.395–1.564)	0.912	1.096 (0.217–5.541)

**Notes.**

* *p* < 0.05, statistically significant.

The prognoses for 100 pairs of patients after exactly matching were evaluated using KM curves (Figs. 2C, 2F). The difference between wedge resection and segmental resection did not achieve statistical significance: for OS, the 5-year survival rates of wedge resection and segmental resection were 90.07% and 94.17% respectively, and the 10-year survival rates of wedge resection and segmental resection were 83.48% and 84.78%, respectively (*P* = 0.320); for LCSS, the 5-year survival rates of wedge resection and segmental resection were 97.89% and 98.75%, respectively, and the 10-year survival rates of wedge resection and segmental resection were 93.67% and 98.75%, respectively (*P* = 0.342).

### Univariate COX regression analyses of TC patients who underwent wedge resection and segmental resection before PSM

Relative risk factors for OS and LCSS were analyzed using a univariate COX regression hazard model (Table 6). The study showed that age was only a prognostic factor for OS and LCSS: HR = 4.141 (95% CI [2.430–7.058], *P* < 0.001) for OS, and HR = 5.428 (95% CI [1.296–22.728], *P* = 0.021) for LCSS. Notably, gender (*P* = 0.093) and tumor size (*P* = 0.682) no longer independently affected the prognoses for OS and LCSS, respectively.

### Subgroup analysis of TC patients who received wedge or segmental resection for OS and LCSS before PSM

**Figure 4:**
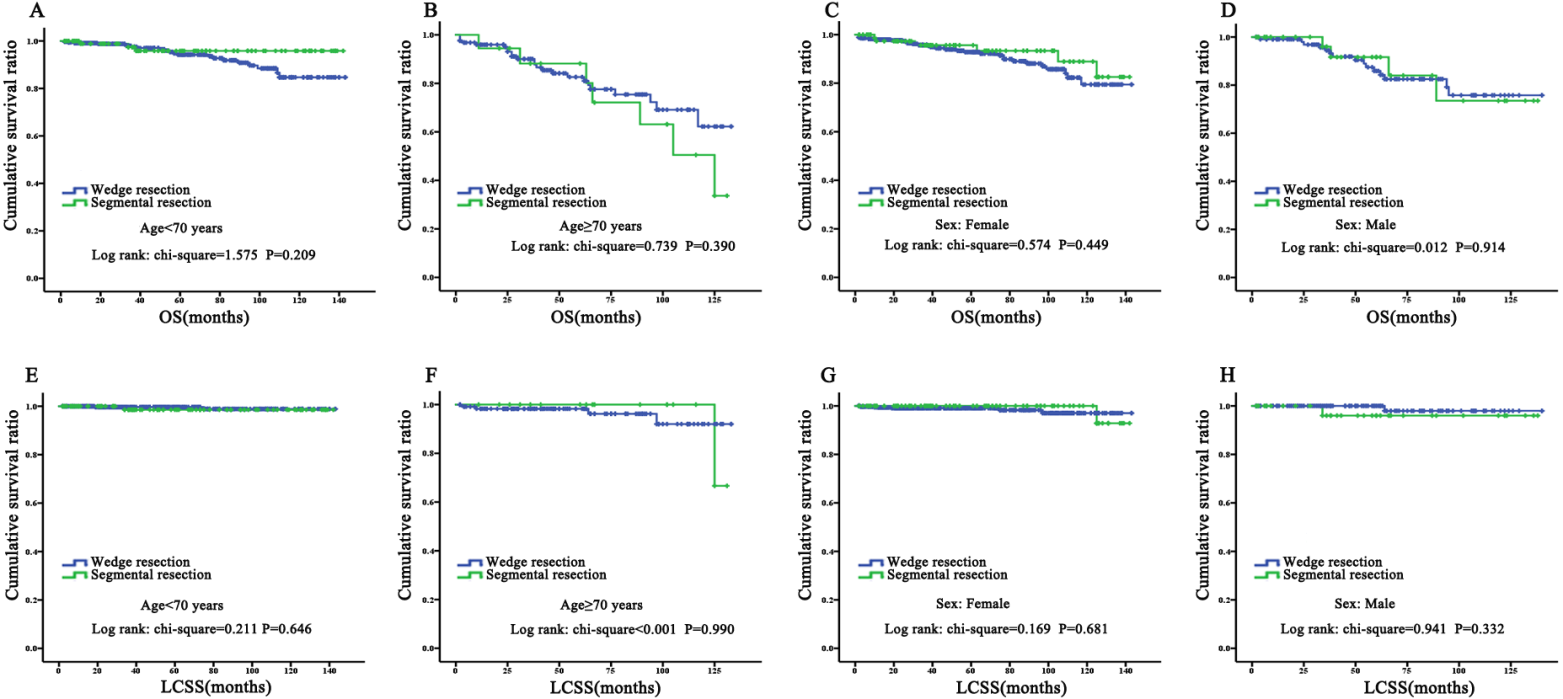
Prognostic analysis for subgroup of age and sex by KM plots. Overall survival and lung cancer specific survival in subgroups of age (<70 and ≥70) and gender (female and male) between wedge resection and segmental resection. There was no significant difference between two surgical options in OS: (A) age < 70; (B) age ≥ 70; (C) female; (D) male. Besides, there was no significant difference between two surgical options in LCSS: (E) age < 70; (F) age ≥ 70; (G) female; (H) male. OS, overall survival; LCSS, Lung cancer-special survival.

**Figure 5:**
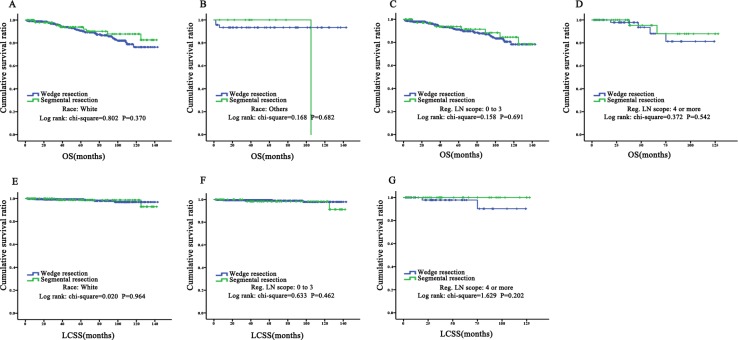
Prognostic analysis for subgroup of race and Scope of Regional Lymph Node by KM plots. Overall survival and lung cancer specific survival in subgroups of race (white and others) and Scope of regional Lymph node (0–3 and ≥3) between wedge resection and segmental resection. There was no significant difference between two surgical options in OS: (A) white; (B) others; (C) 0–3; (D) ≥3. Besides, there was no significant difference between two surgical options in LCSS: (E) white; (F) 0–3; (G) ≥3. No death case was found in Others (subgroup of race) in LCSS so that the KM curves was missing. OS, overall survival; LCSS, Lung cancer-special survival; Scope of Reg. LN Sur., Scope of Regional Lymph Node.

**Figure 6:**
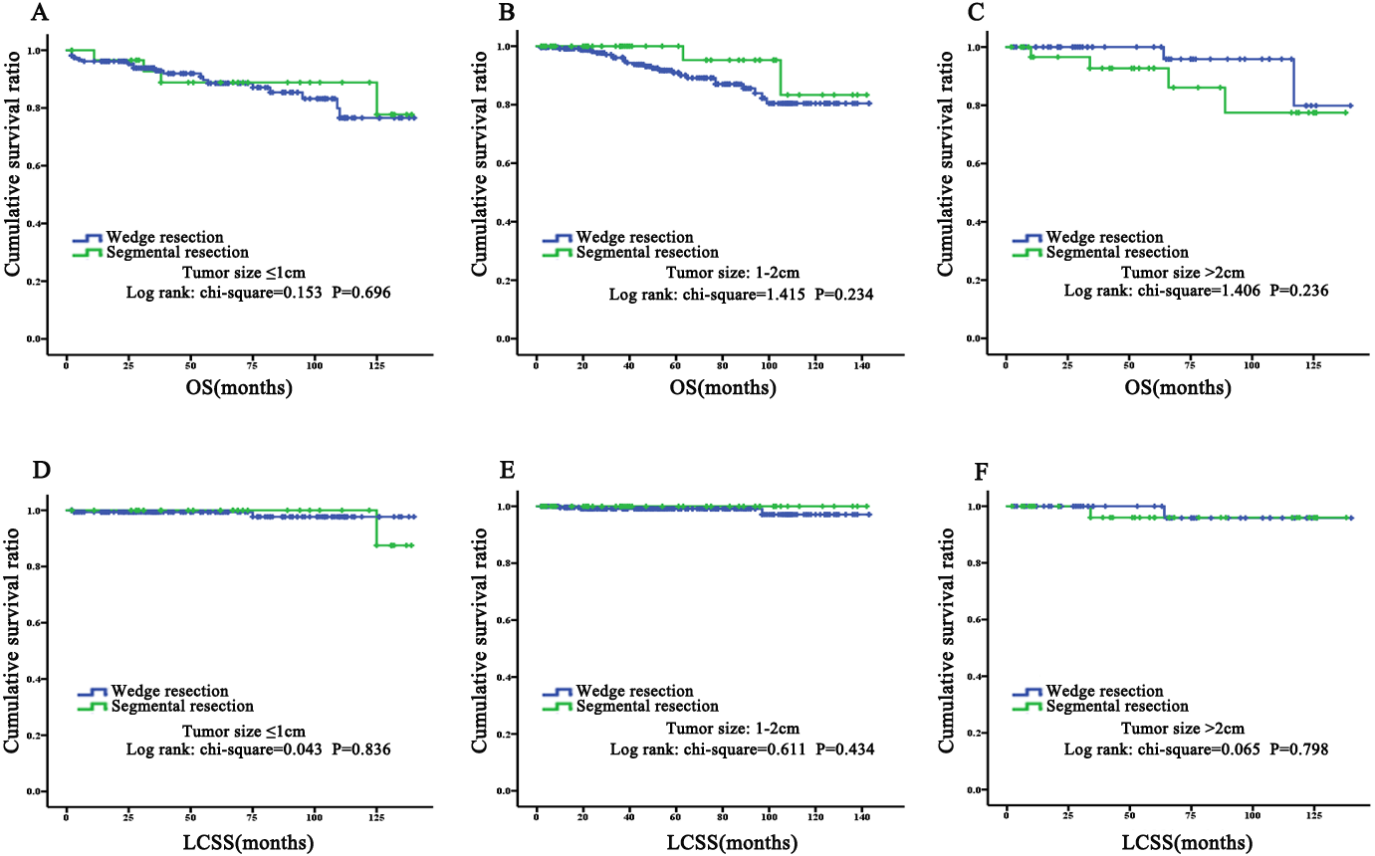
Prognostic analysis for subgroup of tumor siza by KM plots. Overall survival and lung cancer specific survival in subgroups of tumor size (size ≤1 cm, 1 cm 2 cm) between wedger resection and segmental resection. There was no significant difference between two surgical options in OS: (A) size ≤ 1 cm; (B) size >1 cm and size ≤2 cm; (C) size > 2 cm. Similarly, there was no significant difference between two surgical options in LCSS: (D) size ≤ 1 cm; (E) size >1 cm and size ≤ 2 cm; (F) size > 2 cm. OS, overall survival; LCSS, Lung cancer-special survival.

The results showed no statistically significant difference between wedge and segmental resection in any subgroup of age, race, gender, tumor size, or scope of regional lymph node, which was rendered in KM plots (Figs. 4–6). Cox regression analysis for OS also supported that view (Table 7). The results of Cox regression analysis for LCSS are not shown because death was rare after sectionalization.

**Table 7:** Subgroup analyses according to OS and LCSS before PSM.

Variables in the equation	OS	
	*P*	Hazard ratio (95% CI)
Age		
<70y	0.220	0.469 (0.140–1.574)
≥70y	0.393	1.448 (0.619–3.384)
Gender		
Female	0.451	0.712 (0.295–1.721)
Male	0.914	0.941 (0.312–2.837)
Race		
White	0.373	0.719 (0.348–1.485)
Others	0.686	1.601 (0.163–15.704)
Tumor size		
T1 (≤1 cm)	0.696	0.804 (0.269–2.403)
T2 (≤2 cm, >1 cm)	0.249	0.428 (0.101–1.811)
T3 (≥2 cm)	0.254	2.706 (0.489–14.969)
Scope of regional lymph node		
*n* ≤ 3	0.692	0.857 (0.400–1.837)
*n* > 3	0.546	0.592 (0.108–3.245)

## Discussion

The study showed that the prognosis of TC patients was best among those suffering from ADC and SCC. Further univariate and multivariate Cox analysis showed that the surgery option was independent of the prognosis for OS and LCSS in TC patients. As for the impact of surgery type (wedge and segmental resection) on survival, the result was not statistically significant either before or after PSM. In conclusion, the important discovery of this report is that wedge resection is likely to be equal to segmental resection for TC patients at the localized stage.

As the low-grade lung NET, TC has a favorable prognosis, where the 5-year and 10-year survival rate for OS and LCSS were highest compared with NSCLC and AC (Table 1). Some studies also showed that 5-year OS of TC was more than 87 percent (Filosso et al., 2013; Gosain et al., 2018). The excellent prognosis for TC may be attributed to a handful of mitoses, no necrosis, and a low Ki-67 labeling index (Caplin et al., 2015; Rindi et al., 2014). However, other research has indicated that the survival rate for Pulmonary carcinoids (PCs) has dropped for the past 30 years (Gustafsson et al., 2008). In its milder forms, it may still be a major problem increasing mortality and delaying diagnosis. Compared with other lung carcinomas, only 10.4% of primary TC is located in the bronchi, so respiratory symptoms, such as chest infections, cough, hemoptysis, and chest pain are relatively rare (Gustafsson et al., 2008; Caplin et al., 2015). Similarly, lung neoplasms were larger than five cm resected from the TC patients at the localized stage because the symptoms were so mild.

For TC patients who were at the localized stage, age, gender, and surgery option were independent prognostic factors for OS in the multivariate Cox analysis. However, the factor “gender” was replaced by the factor “tumor size” in assessment for LCSS in the multivariate analysis. Same with James W. Vaupel ’s finding, Women live longer than men today (Zarulli et al., 2018). Long life span of female may contribute to the cause differences in analysis of OS. Besides, unlike OS, people were classified as having died from LCSS only when the cause was related to lung cancer. Therefore, gender may not influence the progress of death from TC. The significant difference between the group with no surgery and the group who underwent surgery indicated that surgery was essential for the treatment of TC even though it had low malignancy. In a Cox regression model with time dependence, age was statistically significant (data not shown). The HR increases as patients age.

Like the results of Taher Abu Hejleh et al. (Furqan et al., 2018), research on surgery type showed that TC patients at the localized stage would benefit equally from lobectomy or sublobectomy. However, Taher Abu Hejleh et al. overlooked that sublobectomy contained both wedge and segmental resection. Comparisons between sublobectomy and lobectomy respectively were not done. Thus, superiority of lobectomy cannot be proved by being contrasted with sublobectomy, similar anatomy resection (segmentectomy), or wedge resection.

The emphasis of this research is on whether the benefits of wedge resection can equal those of segmentectomy. The raw data indicated that the choice between the two procedures did not influence the prognosis of patients who underwent sublobectomy. Furthermore, after 1:1 PSM, the gaps between the OS and LCSS survival rate of two surgery procedures closed, providing additional evidence for the uniformity of the benefits from treatment, whether treatment consisted of wedge resection or segmentectomy produced in TC therapy. Besides, the Cox analysis of TC patients who underwent those two surgeries before PSM showed that only age was a relevant prognostic factor for OS or LCSS, and surgery type was no exception. The therapeutic effect of sublobectomy eliminated the impact of factors other than age on prognosis and both two operating methods were effective for TC. Furthermore, this held true for many subgroups of TC patients, including groups that were younger or older, or male or female, verified by the subgroup analyses.

A survey that involved 172 institutions worldwide showed that only 11 percent of participants regarded wedge resection as an appropriate surgical option for peripheral PCs (Caplin et al., 2015). However, the results of this research showed that patients did not benefit more from segmental resection than wedge resection. Compared to the anatomic resection, the operation wound from wedge resection was milder and caused less damage to pulmonary function. Therefore, the results proved that wedge resection, as well as anatomic resection, should be considered a conventional treatment for TC patients at the localized stage. Preserving better pulmonary function and leaving a smaller surgical wound will promote better quality of life for patients undergoing the surgery.

Inevitably, there are several limitations similar to those of most retrospective studies based on the SEER database used for this study. First of all, information on patients, such as complications, recurrence, follow-up treatment received, TMN stage, and so on was not complete. This reduced the level of accuracy and detail of the prognoses. Secondly, the number of cases was limited because the disease is rare. The results of the research will be more convincing when additional cases are studied. Third, this research may include more bias than a prospective study or a randomized trial.

## Conclusion

The wedge resection was a comparable treatment to segmental resection for TC patients at the localized stage.

##  Supplemental Information

10.7717/peerj.7519/supp-1 Table S1 Raw data for 1887 TC patients

10.7717/peerj.7519/supp-2 Table S2 Raw data for SCC TC AT patients in Table 1 and Fig. 1

10.7717/peerj.7519/supp-3 Dataset S1 Raw data for ADC patients in Table 1 and Fig. 1

## Additional Information and Declarations

### Data Availability

The following information was supplied regarding data availability: The raw measurements are available in Table S1, Table S2, and Dataset S1.
